# miR-21-5p Inhibits the Proliferation, Migration, and Invasion of Glioma by Targeting S100A10

**DOI:** 10.7150/jca.84030

**Published:** 2023-06-19

**Authors:** Peng Gao, Haopeng Wang, Huaixu Li, Lei Shu, Zhenyu Han, Shiting Li, Hongwei Cheng, Xingliang Dai

**Affiliations:** 1 Department of Neurosurgery, the First Affiliated Hospital of Anhui Medical University, Hefei, 230022, Anhui, China.; 2 Department of Neurosurgery, Xinhua Hospital, School of Medicine, Shanghai Jiao Tong University, Shanghai 200092, China.; 3 Department of Clinical Medicine, The First Clinical College of Anhui Medical University, Hefei, 230032, Anhui, China.; 4 Department of Medical Imaging Technology, The First Clinical College of Anhui Medical University, Hefei, 230032, Anhui, China.; 5 Department of Neurosurgery, Affiliated Jinling Hospital, Medical School of Nanjing University, Nanjing, 210002, Jiangsu, China.

**Keywords:** S100A10, glioma, miR-21-5p, progression, prognosis.

## Abstract

S100A10, a member of the S100 protein family, is upregulated in multiple human malignancies and plays a key role in regulating tumor progression. This study aimed to reveal the underlying mechanism by which S100A10 in regulates the proliferation, migration, and invasion of glioma. The expression and clinical information data of S100A10 were downloaded from public databases (TCGA, CGGA, and GEPIA2). S100A10 expression levels in glioma tumor tissues and adjacent nontumor tissues were compared by immunohistochemistry (IHC). The functional roles of S100A10 in glioma were assessed by cell counting kit-8 (CCK-8) cell proliferation assay, wound healing assay, transwell assay, and flow cytometry. miRDB and double luciferase assay were used to predict and identify potential S100A10 mRNA-complementary miRNAs, and the roles of miR-21-5p in glioma cell were examined by targeted knockdown or overexpression miR-21-5p in glioma cell lines. We found that S100A10 was overexpressed in glioma tissues and predicted a worse prognosis. S100A10 knockdown significantly inhibited glioma cell proliferation, invasion, and migration. Furthermore, we demonstrated that miR-21-5p inhibits glioma proliferation, migration, and invasion by targeting S100A10. This study showed S100A10 was a new prognostic predictor among glioma patients and provided new insights into the pathogenesis of gliomas, suggesting that miR-21-5p /S100A10 axis may serve as a valuable therapeutic target for glioma.

## Introduction

Glioma is an aggressive central nervous system tumor with a poor prognosis^1^, accounting for about 50% of primary intracranial tumors^2^. The World Health Organization (WHO) has classified glioma into Ⅰ-Ⅳ grades. Among these, grade Ⅲ-Ⅳ (also called high-grade glioma) has a very poor prognosis^3^, and its standard treatment methods are limited to the maximum safe surgical excision, chemotherapy, and radiotherapy. Although with adjuvant temozolomide and standard radiotherapy after maximum safe resection, the median survival time of glioma patients is still less than 2 years. Thus, it urgently needs to find a method to identify the prognosis of glioma and slow its progression^4^.

S100A10 also known as P11, CAL1L (Calpactin I, light chain), CLP11 (Calpactin I, P11 subunit), or ANX2LG (Annexin II Ligand)^5^, It's a member of the S100 protein family, located on the plasma membrane. It usually forms a heterotetramer complex with annexin A2, which contains two annexin A2 and two S100A10 subunits. S100A10 is a plasminogen receptor, and the heterotetramer complex formed by S100A10 exerts functions via the plasminogen activation pathway. The heterotetramer complex binding to plasminogen can activate tissue-type plasminogen activator (t-PA) and urokinase-type plasminogen activator (u-PA), and convert plasminogen to plasmin, which can degrade fibrin and activate matrix metalloproteinase (MMPs), and finally, promoting the degradation of extracellular matrix (ECM). Thus, S100A10 plays a key role in the biological processes of tumor. Such as proliferation, migration, invasion and so on. The up-regulated expression of S100A10 is involved in a variety of tumor malignant characteristics, indicating that S100A10 may be a potential tumor prognostic marker^5^-^7^. Some studies have searched the expression of S100 protein in the renal lesions after surgical resection of non-cancerous areas and renal cell carcinoma by quantitative polymerase chain reaction (qPCR) and found that S100A10 was expressed in the renal cell carcinoma, but was not found near the carcinoma^8^. Another study found that the expression of S100A10 could be induced by GAS6/AXL signal, and promote angiogenesis and invasion ability of renal clear cell cancer cells^9^; One study has found that the high expression of S100A10 in gallbladder carcinoma is associated with poor prognosis^10^. A study has also found that high expression of S100A10 in lung squamous cell carcinoma cells, especially near the stroma, plays an important role in tumor progression^11^. Although S100A10 has been studied in a variety of tumors, it has not been reported in gliomas. Recent studies have found that S100A10 is also involved in regulating nerve cell function^12^, ^13^.

microRNAs (miRNAs) are single-stranded, non-coding RNA molecules, with 19-22 nucleotides in length, and they regulate approximately 30-50% of human genes via inducing degradation or translational inhibition of their target mRNAs. They are widely involved in the progression of tumors, becoming a hotspot in the study of tumor pathogenesis^14^-^16^. miRNA-21, as a member of microRNAs, has been found to play a key role in tumor regulation. For example, miR-21-5p induces the proliferation of non-small cell lung cancer cells by targeting transforming growth factor b-induced protein (TGFBI)^17^, and overexpression of miR-21-5p promotes the proliferation and invasion of colon cancer cells by targeting neural cell adhesion molecule L1(CHL1)^18^. In addition, Alja Zottel found that the expression of miR-21-5p was significantly higher in glioblastoma tissue than that in low-grade glioma and control brain tissue^15^. However, the relationship between miR-21-5p and S100A10 and how it affects glioma progression remains unclear.

In this study, we first revealed that the S100A10 protein expression was regulated by miR-21-5p. We analyzed the correlation between the S100A10 expression with patients' clinical outcomes in the Cancer Genome Atlas (TCGA) data cohort and the relationship between different molecules. The S100A10 biological functions of proliferation, apoptosis, clonal formation, and migration of glioma cells were further investigated *in vitro*. The study aimed to illustrate the correlation between the S100A10 protein expression and the pathological grade of glioma, as well as its biological function in gliomas.

## Materials and methods

### TCGA data

We used a cohort (n=597) from TCGA in this study. Gene expression data, clinical significance, and follow-up information of the patients were obtained from the TCGA data portal (http://tcga-data.nci.nih.gov/tcga/) to identify the differential expression of S100A10 and its prognostic value. Data was collected from the interactive analysis of gene expression profiles (http://gepia.cancer-pku.cn) to explore the correlation between the expression of S100A10 and the overall survival in glioma patients in TCGA and analyze the effect of S100A10 expression and glioma molecular changes (EGFR, MYCN, MGMT, PTEN) on the survival rate of glioma patients. The inclusion and exclusion criteria of data for the further analysis were as follows. The inclusion criteria: 1) Data of gliomas including Low grade gliomas (LGG), High grade gliomas (HGG); 2) The gene expression of fragments per kilobase million (FPKM) were normalized in transcriptome. The exclusion criteria: 1) Survival time is less than or equal to 30 days; 2) Clinical data is null or information is not clear; 3) Annotation information is not matched with the samples information. 4) Duplicate data.

### Cell lines

Human glioma cell lines U87, U251, and U118 were purchased from the Chinese Academy of Sciences, Shanghai Cellbank (Shanghai, China). Human glial cell line NHA was purchased from BeNa Culture Collection (BNCC, Beijing). Human glioma cell lines U343, SNB19, and SHG44, and glial cells G005 were established and cultured in our laboratory. The cells were maintained in Dulbecco's modified Eagle's medium (DMEM, Invitrogen, Valencia, CA) supplemented with 10% fetal bovine serum (Invitrogen, Valencia, CA) in a humidified incubator with 5% CO_2_ at 37 ^o^C, and were passaged twice a week.

### Clinical specimens

The collection of samples and clinical information in this study was approved by the Ethics Committee of the First Affiliated Hospital of Anhui Medical University, and written informed consent was obtained from each patient. At least two neuropathologists diagnosed each sample in this study as diffuse glioma (WHO Ⅱ, Ⅲ, and Ⅳ). Seventy-nine consecutive patients who were diagnosed with primary glioma (WHO Ⅱ27, Ⅲ 14, Ⅳ 38) between September 2018 to September 2019 at Department of Neurosurgery, the First Affiliated of Anhui Medical University were collected in this study. No pretreatment radiation and chemical therapy were administered in any of these patients. Matched para-carcinoma tissues were obtained from areas away from the tumor, to avoid tumor field effect. Normal brain tissues were obtained from six brain injury patients.

### RNA Extraction, Reverse Transcription, and qPCR Analysis

Total RNA was extracted using TRIzol reagent (Invitrogen), and RNA concentration was measured using the NanoDrop 2000 spectrophotometer (Thermo Fisher Scientific, Inc.). Reverse transcription of total RNA was performed using HiScript II Q RT SuperMix for qPCR (Vazyme, Nanjing, China), with random primers at of 55˚C for 15 min, followed by 85˚C for 5 sec. Quantitative real-time PCR(qPCR) was performed using SYBRs Green PCR Master Mix (Vazyme) on Roche instruments (Applied Biosystems). The following primer sequences were used for qRT-PCR: The primer of human S100A10 gene was CCGCACGTACTAAGGAAGGC and TCATGGTTTCCATGGCGTGT. Glyceraldehyde-3-phosphate dehydrogenase (GAPDH) was used as the endogenous control, and its primer was AGGTCGGTGTGAACGGATTTG and TGTAGACCATGTAGTTGAGGTCA; The primer of miR-21-5p was TCGCCCGTAGCTTATCAGACT and CAGAGCAGGGTCCGAGGTA. U6 snRNA was used as negative control and its primer was CGCTTCGGCAGCACATATAC and TTCACGAATTTGCGTGTCATC. The following thermocycling conditions were used for qPCR: an initial denaturation step at 95℃ for 5 minutes, followed by 40 cycles of denaturation at 60℃ for 15 seconds. A melting curve analysis of each sample was used to check the specificity of amplification, and each sample was assayed in triplicate. The 2^-ΔΔCt^ method^19^ was used as a relative quantification measure of differential expression and normalized to the internal reference genes U6 and GAPDH.

### Cell Transfection

Transfection of U87 and U251 cells was performed using siRNA-mate reagent (GenePharma, Shanghai, China). S100A10 small intefering RNA vector ((S100A10-Homo-514) sense (5'-3') CUCAAAUGGAACACGCCAUTT, anti-sense (5'-3') AUGGCGUGUUCCAUUUGAGTT), miR-21-5p mimics (sense (5'-3'): UAGCUUAUCAGACUGAUGUUGA and anti-sense (5'-3'): AACAUCAGUCUGAUAAGCUAUU), miR-21-5p inhibitor (UCAACAUCAGUCUGAUAAGCUA) were designed and synthesized by Shanghai GenePharma Co., Ltd (Shanghai, China). and control miRNA was used as a negative control. The level of gene silencing was detected by qPCR within 48h, and the protein expression was detected by western blotting within 72h.

### Tissue Microarray and HE Staining

The collected glioma tissues, para-carcinoma tissues, and normal brain tissues were used to fabricate tissue microarray for HE and IHC stainings. Tissue samples embedded in paraffin were sectioned, deparaffinized, and subjected to antigen retrieval performed in citrate buffer (pH 6.0). Slides were incubated at 4 ℃ overnight with the S100A10 antibody (1:500; Cat. no. 0041696; Proteintech, Wuhan, China) and then with a secondary HRP-conjugated antibody (Proteintech) at 37^ o^C. Sections were stained with 3-diaminobenzidine (DAB) for 2 min. All pathological sections were scanned with high-resolution images via full-field digital slice scanner (Pannoramic MIDI, Hungary).

### Cell Proliferation Assay

The cell proliferation was evaluated by CCK8 kit (Beyotime, Shanghai, China). The seeding of cells in their log growth phase (4 × 10^3^ cells/well) was done and maintained in cell culture plates (96-well) and incubated at indicated times. After incubation, CCK8 solution (containing 100μl culture solution and 10μl CCK8 reagent) was added to each well and incubated for an additional 1 hour at 37℃ and then placed under 450 nm wavelength of the microplate reader, testing the optical density (OD) value of each well and carrying out proliferation curve.

### Flow Cytometric Analysis of Cell Apoptosis

The cell apoptosis was tested by cell apoptosis detection kit (BestBio, Shanghai, China). Cells were digested into single cells with trypsin (without EDTA) and washed twice with ice-cold PBS. Annexin V staining was carried out using the cell apoptosis detection kit according to the manufacturer's instructions. After 15 min incubation in a dark at 4℃ and then added PI staining gently mix and incubate at 4℃ for 5min, the cells were immediately analyzed by flow cytometer.

### Transwell Assay

Cell invasion assay was performed using Transwell cell culture inserts with 8 μm pores (Corning, MA). Matrigel (BD, CA) coating membrane inserts was added to the inserts for 2 h before cells were plated into inserts according to the manufacturer's instruction. 2×10^5^ cells were resuspended in serum-free media and loaded into 8 μm membrane inserts and placed over media containing 20% fetal bovine serum. After incubation for 24 h, the non-invasion cells on the upper chamber were erased by cotton swab. The invasion cells adhered to the membrane lower chamber were fixed with 4% paraformaldehyde for 30 min, stained with hematoxylin for 30 min and then number of cells was counted under a microscope in 3 random optical fields.

### Dual‑luciferase reporter assay

miRDB (http://mirdb.org/) was used to predict the target gene and potential binding sites of S100A10. Cells were seeded into 24‑well plates at a density of 2×10^4^ cells/well and cultured with DEME medium containing 10% fetal bovine serum. After incubated into 50-60% confluence, cells were subsequently co‑transfected with miR‑21‑5p mimics or inhibitor, and the pmirGLO luciferase vector (Promega Corporation) containing wild type (WT) or mutant (MUT) 3'‑UTR of S100A10 using Lipofectamine 2000 (Invitrogen; Thermo Fisher Scientific, Inc.). They were harvested for luciferase detection with the dual-luciferase reporter assay system (Promega, Madison, WI, USA) according to the manuals of the manufacturer's protocol. The Firefly luciferase activity was normalized to Renilla luciferase activity.

### Western Blotting

This part refers to our previous experimental methods^20^. Briefly, Total cellular proteins were lysed with a radio-immunoprecipitation assay (RIPA). The concentrations of the proteins were determined using the bicinchoninic acid (BCA) assay method and equal concentrations of the proteins were loaded on SDS-PAGE gel (10%). Thereafter, the samples were transferred onto polyvinylidene difluoride (PVDF) membranes and blocked with 5% skimmed milk solution for 1 hour. Next, membrane was incubated with S100A10 rabbit monoclonal antibody (1:1000; Cat. no. 0041696; Proteintech, Wuhan, China) or GAPDH rabbit polyclonal antibody (1:5000; Cat. no. 10494-1-AP; Proteintech, Wuhan, China) at 4°C overnight. After washing with PBST for 4 times, the membranes were incubated with horseradish peroxidase-linked secondary biotinylated antibodies for 1.5h at room temperature. Following washing with PBST for 4 times, the immunoreactive bands were observed using the enhanced chemiluminescence (ECL) reagent (Vazyme, Nanjing, China), and the images were recorded in the Gel Imaging System. The relative S100A10 expression levels were calculated by the Gel-Pro-Analyzer (Tanon, Shanghai, China).

### Statistical Analysis

All statistical analyses were performed using GraphPad Prism version 8.0.2 (GraphPad Software Inc, La Jolla, CA, USA). Continuous data were expressed as mean ± standard deviation (SD). The significant difference was determined using Student's t-test. *P<0.05* was considered to be statistically significant.

## Results

### Database analysis suggested that high expression of S100A10 in glioma tissues predicted worse prognosis

**Figure 1:**
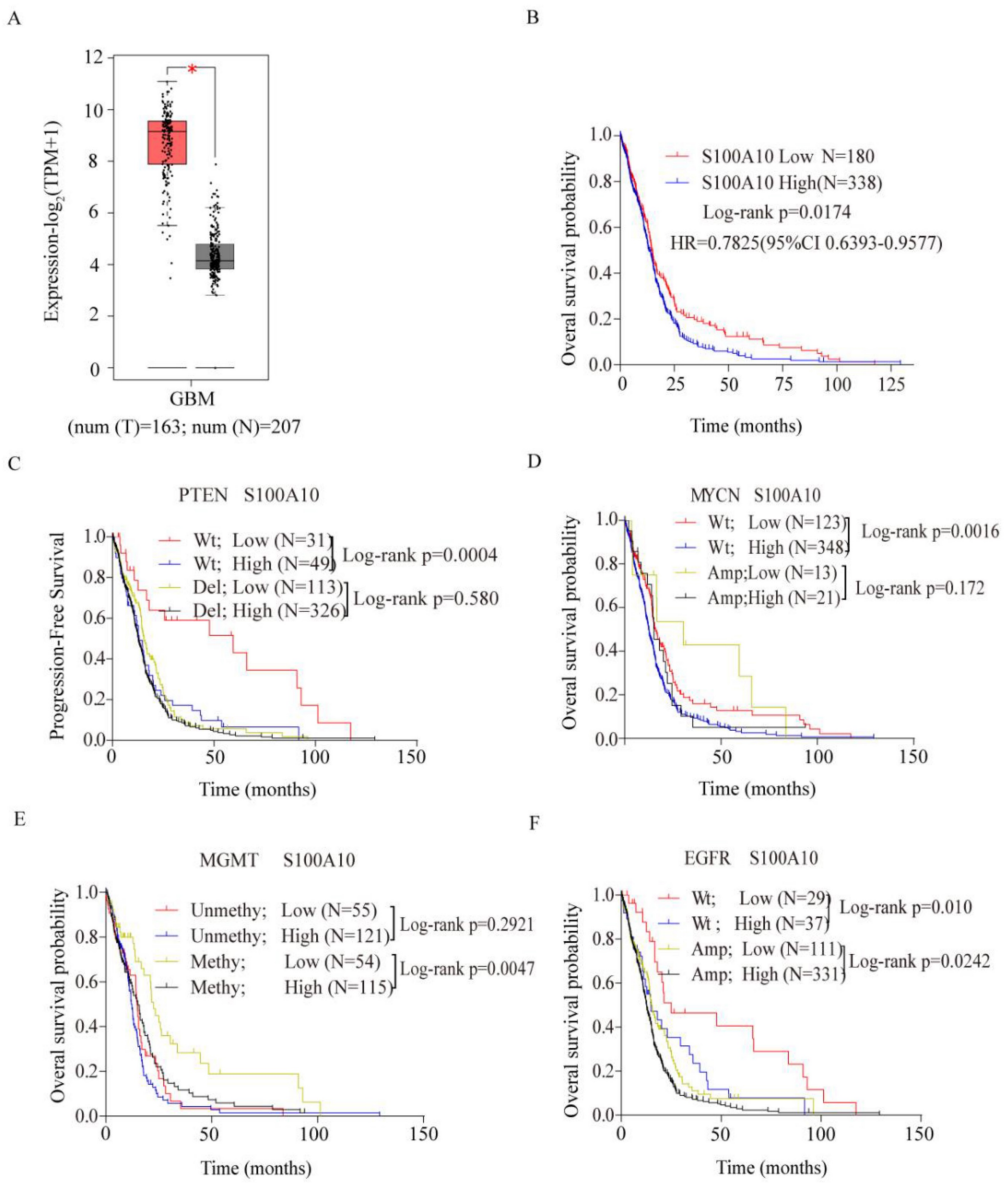
** Database analysis suggested that S100A10 was highly expressed in glioma and predicted a worse prognosis. (A)**: S100A10 expression in glioma and normal brain tissue was analyzed by TCGA database; **(B)**: The relationship between S100A10 expression and overall survival of glioma patients was analyzed in the TCGA database; **(C)**, **(D)**, **(E)** and **(F)**: TCGA and CGGA databases analyzed the relationship between S100A10 expression and other genetic changes on the overall survival probability and progression-free survival probability of glioma patients, including PTEN**(C)**, MYCN**(D)**, MGMT**(E)**, EGFR**(F)**. (WT, Wild type; Del, Deletion; Amp, Amplification; Methy, methylation; Unmethy Unmethylation; *, P<0.05)

The expression level of S100A10 in glioma and normal tissues was analyzed in TCGA database. The detailed clinic parameters of enrolled patients from the TCGA database were summarized in Table 1. It was found that S100A10 showed high expression in glioma compared with normal tissues (Figure 1A). Then we analyzed the prognosis of glioma patients with S100A10 expression in TCGA database, and found that high S100A10 expression showed a poor overall survival rate in glioma patients (Figure 1B). To further explore the expression profile of S100A10 gene, we analyzed the effects of S100A10 expression and other common genetic changes on progression-free survival and overall survival in glioma, including PTEN, MYCN, MGMT, and EGFR. Results suggest that the high expression of S100A10 in the presence of wild-type PTEN, wild-type MYCN, and methylated MGMT gene predicted poor progression-free survival or overall survival, while there was no significant difference in the prognosis of patients in the presence of wild-type and amplified EGFR genes (Figure 1C, 1D, 1E, 1F). The above results indicate that S100A10 is highly expressed in glioma, which predicts a worse prognosis, and had a close relationship with wild-type PTEN, wild-type MYCN, and methylated MGMT gene changes.

### S100A10 was highly expressed in glioma tissue and positively correlated with glioma grade

**Figure 2:**
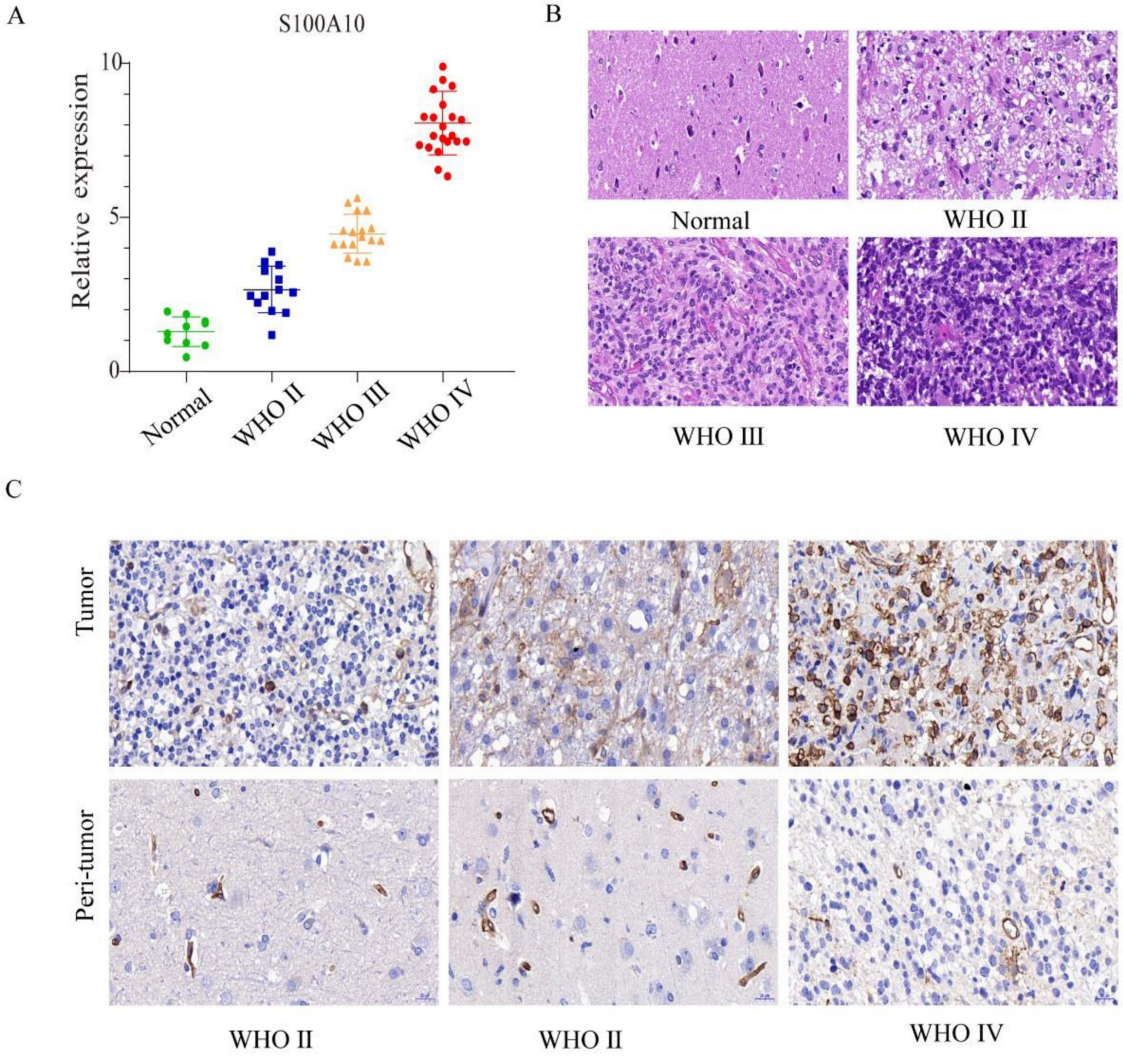
** S100A10 was highly expressed in glioma tissue and positively correlated with glioma grade. (A)**: qPCR detects S100A10 mRNA expression in the normal brain tissue, WHO Ⅱ, WHO Ⅲ, and WHO Ⅳ grade glioma tissue. **(B)**: HE staining in the normal brain tissue, WHO Ⅱ, WHO Ⅲ and WHO Ⅳ grade glioma tissue; **(C)**: Immunohistochemical staining in the normal brain tissue, WHO Ⅱ, WHO Ⅲ and WHO Ⅳ grade glioma tissue.

The expression of S100A10 mRNA was analyzed by qPCR in different grade glioma (WHO Ⅱ, Ⅲ and Ⅳ) and normal brain tissue samples. The results showed that the mRNA transcription level of S100A10 increased with the elevation of glioma grade (Figure 2A). Pathological HE staining and immunohistochemistry indicated that with the increase of glioma grade, tissue had evident atypia and deeper stain. The number of cytoplasmic positive marker cells positively correlated with the glioma grades (Figure 2B, 2C). Generally, these results indicated that S100A10 has high expression in high grade glioma.

### Down-regulation of S100A10 significantly inhibited proliferation, migration, and invasion and promoted apoptosis of glioma cells

**Figure 3:**
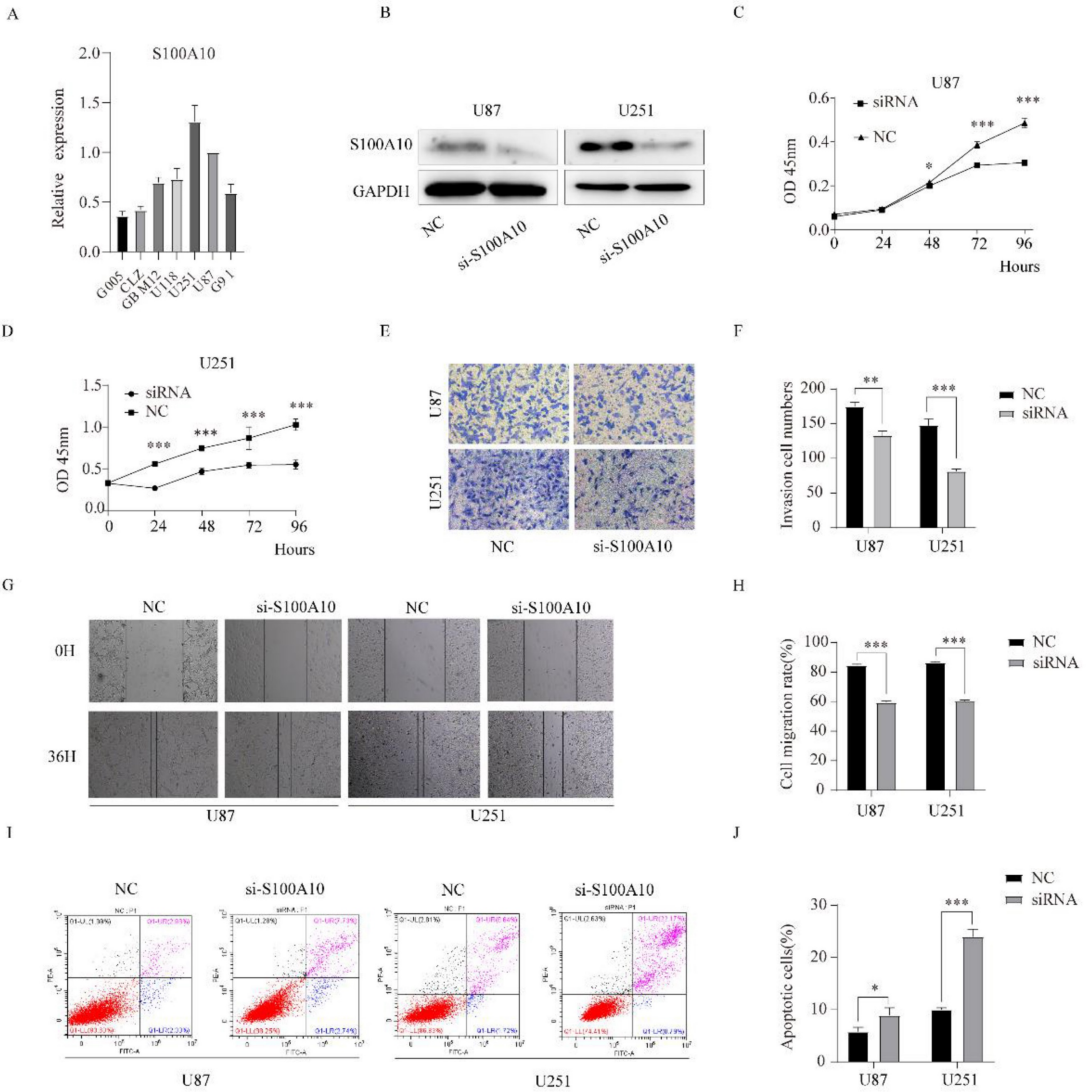
** Downregulation of S100A10 significantly inhibited proliferation, migration, and invasion of glioma cells, and promoted apoptosis of glioma cells. (A)**: qPCR detects S100A10 mRNA expression in normal glioma cell G005, glioma stem cell G91 and glioma cell lines CLZ, GBM12, U118, U87 and U251; **(B):** siRNA of S100A10 was used to down-regulate the expression of S100A10, Western Blotting was used to verify the interference efficiency; **(C)** and **(D)**: CCK-8 proliferation assay was used to detect the proliferation ability of U87 and U251 cells after interfering with S100A10 expression. **(E)** and **(F)**: Transwell invasion assay was used to test the invasion ability of U87 and U251 cells after interfering with the expression of S100A10; **(G)** and **(H)**: Cell scratch assay was used to detect the migration ability of U87 and U251 cells after interfering with S100A10 expression in 36 hours; **(I)** and **(J)**: Flow cytometry was used to detect the changes of apoptosis rate in U87 and U251 cell populations after interfering with S100A10 expression. (NC, negative control; siRNA, si-S100A10; *, P<0.05; **, P<0.01; ***, P<0.001)

The transcription level of S100A10 mRNA in G005, CLZ, GBM12, U118, U251, and U87 cell lines was detected by qPCR. The results showed that the transcription level of S100A10 in glioma cell lines was significantly higher than that in normal glioma cell line G005. In that, U87 and U251 cell lines were the most significantly increased (Figure 3A).

siRNA was used to knockdown the expression of S100A10 in U87 and U251 cell lines, and Western blotting confirmed that the expression of S100A10 in U87 and U251 cells was significantly decreased after transfection of siRNA (Figure 3B). CCK-8 proliferation assay showed that the downregulation of S100A10 could significantly inhibit the proliferation of U87 and U251 cell lines (Figure 3C, 3D). Transwell invasion assay showed that knocking down the expression of S100A10 significantly reduced the invasion ability of U87 and U251 cell lines (Figure 3E, 3F). Cell scratch assay showed that downregulation of S100A10 significantly reduced the migration ability of U87 and U251 cell lines (Figure 3G, 3H). Flow cytometry showed that the apoptosis rate of U87 and U251 cells significantly increased compared with the control group (Figure 3I, 3J). These results indicate that the downregulation of S100A10 expression can significantly inhibit the proliferation, migration, and invasion of glioma cells, and promote the apoptosis of glioma cells.

### miR-21-5p directly targets S100A10 and reduces its expression in glioma cell lines U87 and U251

**Figure 4:**
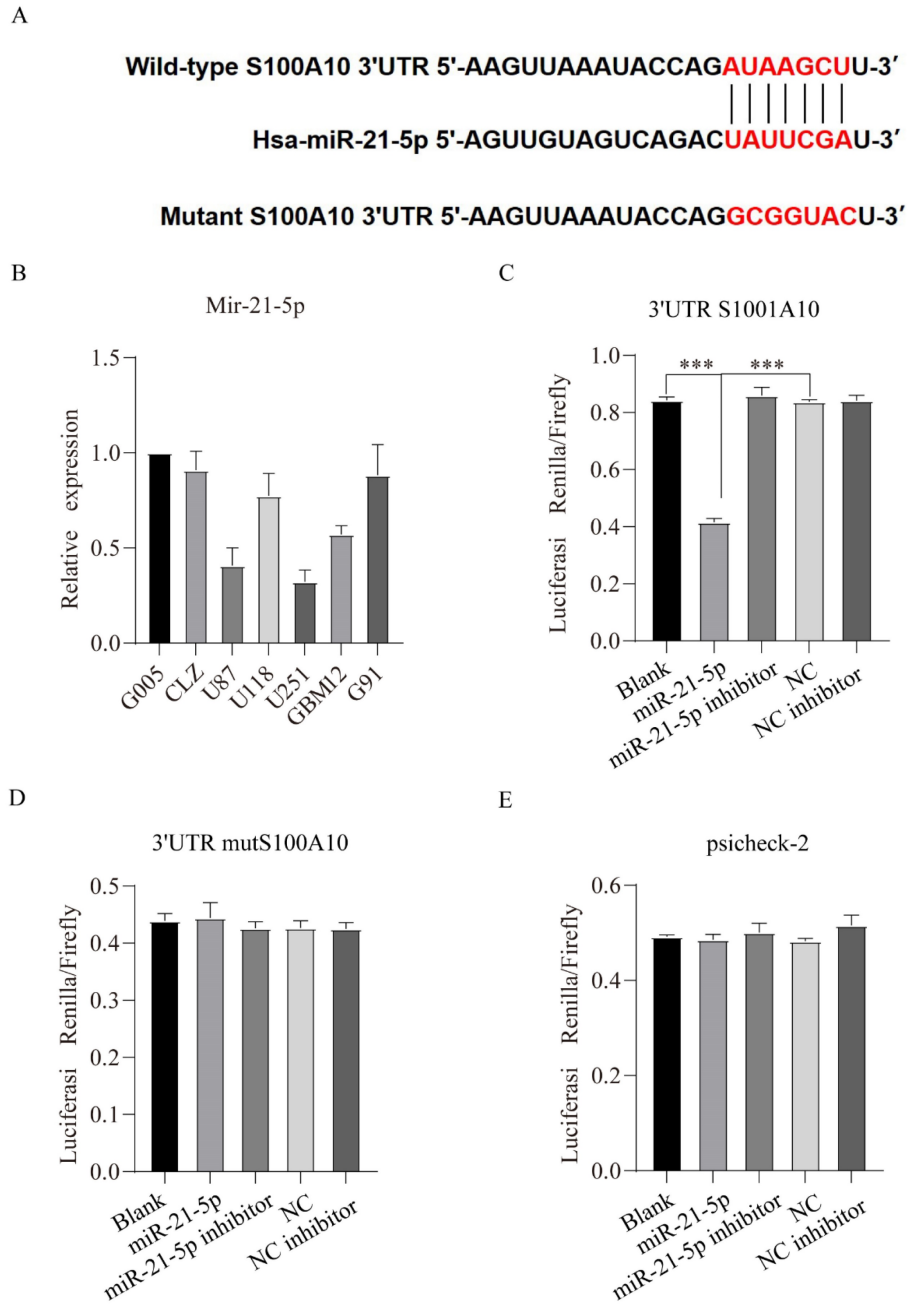
** miR-21-5p directly targets S100A10 and reduces its expression in glioma cell lines U87 and U251. (A)**: Predicted binding sites of miR-21-5p at 3'UTR of S100A10; **(B)**: qPCR was used to detect the expression of miR-21-5p in normal glioma cells, glioma stem cells, and glioma cell lines; **(C)**, **(D)** and **(E)**: Luciferase activity was detected in U87 cell lines with 3'UTR S100A10 gene, MUT 3'UTR S100A10 plasmid, and empty vector psicheck-2, respectively. (NC, negative control; ***, P<0.001)

**Figure 5:**
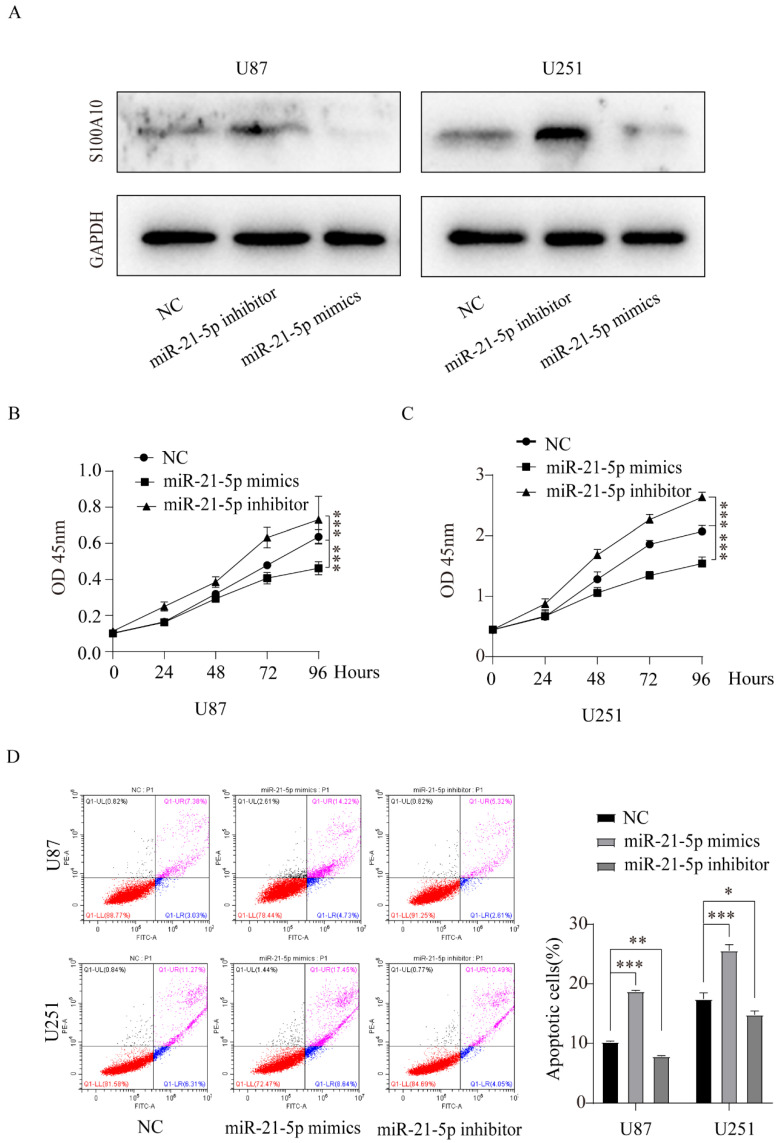
** miR-21-5p regulates the proliferation and apoptosis of glioma cells. (A)**: Western Blotting was used to detect the effects of miR-21-5p mimics and inhibitor on S100A10 expression of U87 and U251 glioma cells; **(B)** and **(C)**: CCK-8 proliferation assay was performed to detect the effects of miR-21-5p mimics and inhibitor on the proliferation of U87 and U251 glioma cells; **(D)**: Flow cytometry assay to detect the effects of miR-21-5p mimics and inhibitor on apoptosis of U87 and U251 glioma cells (NC, negative control; *, P<0.05; ** P<0.01; ***, P<0.001)

miRDB online database (http://mirdb.org/) was used predict regulatory miRNAs of S100A10, and the results showed that the 3'UTR of S100A10 contained binding sites of miR-21-5p, in other words, miR-21-5p is a potential regulatory miRNA of S100A10 (Figure 4A). qPCR results showed that the expression of miR-21-5p was declined in U87 and U251 cell lines with the relatively high expression of S100A10 (Figure 4B). To confirm that S100A10 is the target of miR-21-5p, 3'UTR S100A10 gene, mut 3'UTR S100A10 plasmid and empty vector psicheck-2 were transfected into U87 cell line, respectively, and the luciferase activity was detected. The results showed that, compared with blank group, miR-21-5p could affect the change of fluorescence activity by binding 3'UTR of S100A10 gene (Figure 4C), and there was no statistical difference between miR-21-5p inhibitor group, blank group, and NC inhibitor group; In the experiment which transfected with mut 3'UTR S100A10 plasmid, there was no statistically significant difference between the miR-21-5p group, blank groups, and NC groups, indicating that miR-21-5p couldn't affect the fluorescence activity by binding the 3'UTR of mut S100A10 gene, the binding site was complete mutated (Figure 4D). In the experiment which transfected with empty vector psicheck-2, there was no statistically significant difference between mir-21-5p group and Blank group, indicating that miR-21-5p couldn't affect the fluorescence activity by binding psicheck-2, and there is no binding site (Figure 4E). These results confirmed the binding between mir-21-5p and the 3'UTR of S100A10. Western blotting results also showed that miR-21-5p overexpression significantly reduced the expression of S100A10 protein, and the inhibition of miR-21-5p expression significantly increased the expression level of S100A10 protein in U87 and U251 cell lines (Figure 5A).

### miR-21-5p regulates the proliferation and apoptosis of glioma cells

To further study the role of miR-21-5p in the pathogenesis of glioma, we performed CCK-8 experiment to measure the proliferation changes in U87 and U251 glioma cell lines transfected with miR-21-5p mimics and miR-21-5p inhibitor. The results showed that compared with the control group, miR-21-5p overexpression inhibited the proliferation of U87 and U251 cell lines, while miR-21-5p down-regulation promoted their proliferation (Figure 5B, 5C). Flow cytometry was used to study the effect of miR-21-5p on apoptosis of glioma cell lines U87 and U251, and the results showed that compared with the control group, miR-21-5p overexpression promoted the apoptosis of U87 and U251 cell lines, while inhibition of miR-21-5p expression reduced the apoptosis of U87 and U251 glioma cells (Figure 5D). These results suggest that miR-21-5p regulates the proliferation and apoptosis of glioma.

### S100A10 is regulated by miR-21-5p and affects the invasion and apoptosis of glioma

**Figure 6:**
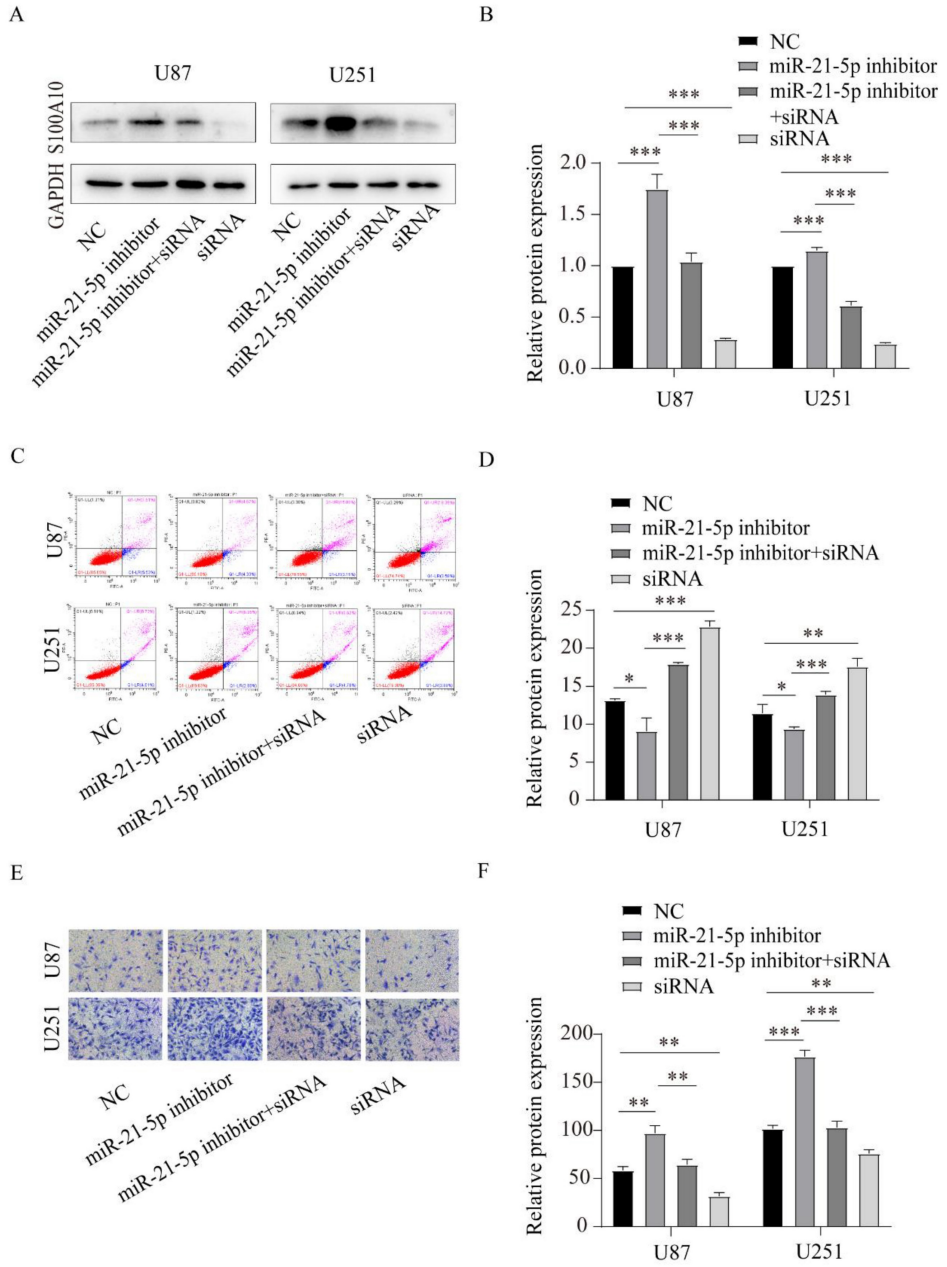
** S100A10 is regulated by miR-21-5p in the invasion and apoptosis of glioma. (A)** and **(D)**: Western Blotting detected S100A10 expression of adding miR-21-5p inhibitor, miR-21-5p inhibitor+siRNA, and siRNA in U87 and U251 glioma cells; **(C)** and **(D)**: Flow cytometry assay was used to detect the effects of miR-21-5p inhibitor, miR-21-5p inhibitor+siRNA and siRNA on apoptosis of U87 and U251 glioma cells. **(E)** and **(F)**: Transwell assay detected the effects of miR-21-5p inhibitor, miR-21-5p inhibitor+siRNA, and siRNA on the invasion ability of U87 and U251 glioma cells. (NC, negative control; siRNA, si-S100A10; P<0.05; ** P<0.01; *** P<0.001)

To confirm that S100A10 was regulated by miR-21-5p to affect glioma invasion and apoptosis, we reversed the expression of S100A10 which was overexpressed by miR-21-5p inhibitor via co-transfecting siRNA of S100A10 in U87 and U251 cell lines. Western blotting assay was used to confirm the transfection effect, and the results showed that the S100A10 protein expression was overexpression by miR-21-5p inhibitor and could be reversed after adding siRNA of S100A10 (Figure 6A, 6B). Flow cytometry showed that adding siRNA-S100A10 significantly reversed the inhibitory effect of miR-21-5p inhibitor on apoptosis of U87 and U251 cells (Figure 6C, 6D). Similarly, Transwell invasion assay showed that the addition of si-S100A10 significantly reversed the promoting effect of miR-21-5p inhibitor on U87 and U251 cell invasion (Figure 6E, 6F). These results suggest that miR-21-5p suppressed glioma cell invasion and apoptosis by targeting S100A10.

## Discussion

Glioma has the characteristics of fast growth, easy metastasis and easy recurrence. The cause of the hard cure of glioma is closely related to these biological characteristics. Therefore, it is of great clinical value to find molecular targets and prognostic indicators that can target the characteristics of glioma cells.

Previous studies have found that S100A10 protein is highly expressed in malignant tumors. Through promoting the degradation of extracellular matrix by converting plasminogen to plasminase, and then activating matrix metalloproteinases, S100A10 can regulate the proliferation, invasion, migration, and angiogenesis of malignant tumors^5^, ^6^. For example, A study find that S100A10 is a prevalent expression in the most invasive anaplastic thyroid cancer, which suggests that S100A10 plays a key role in the progression of thyroid cancer^21^. Other studies have found that S100A10 mRNA and protein are overexpressed in human pancreatic tumors compared with normal ductal and non-ductal stroma in pancreatic cancer, and S100A10 gene knockout reduces surface plasminogen.

In this study, we analyzed the difference in S100A10 gene expression, prognosis, and survival rate in glioma and normal brain tissues by bioinformatics. The results showed that the expression of S100A10 in glioma tissues was higher than that in normal tissues. The expression of S100A10 increased with the grade of glioma, but the survival rate and prognosis of glioma patients declined. This is consistent with our tissue chips and clinical statistics. At present, the prognosis of glioma patients cannot be simply determined by pathological grade. Molecular features such as PTEN deletion, EGFR amplification, MYCN amplification, MGMT methylation, etc. play an important role 22. Our analysis results found that high expression of S100A10 predicted a poor prognosis in the presence of wild-type PTEN, wild-type MYCN, and methylated MGMT gene, while there was no significant difference in prognosis in the presence of wild-type and amplified EGFR genes. These results suggest that S100A10 is overexpressed in gliomas and that its overexpression predicts a worse prognosis and is closely associated with common molecular changes. S100A10 plays an important role in tumor progression and can be used as a prognostic factor.

As aforementioned, S100A10 regulated the proliferation, invasion, migration, and angiogenesis of malignant tumors through the plasminogen activation pathway. Our study found that the knockdown of S100A10 could significantly inhibit the proliferation, migration, and invasion of glioma cells in both U87 and U251 glioma cell lines. These data indicate that S100A10 also plays a key role in regulating the proliferation, migration, and invasion ability of gliomas, and with the higher expression of S100A10, the proliferation, invasion, and invasion ability of gliomas are increased. Abnormal apoptosis is also an important feature of cancer cells, and tumor growth depends on the balance between proliferation and apoptosis^23^. Our flow cytometry data showed that knocking down the expression of S100A10 could significantly increase the ratio of early and late apoptosis of glioma cells, suggesting that knocking down the expression level of S100A10 could promote the apoptosis of glioma cells.

miRNAs play a key role in the progression of malignant tumors, they are considered potential anti-tumor therapeutic targets^24^, ^25^. Our current research results show that over-expression of miR-21-5p can decrease the proliferation ability and increase the apoptotic ability of gliomas, while inhibition of miR-21-5p can increase the proliferation ability and decrease the apoptotic ability of gliomas, indicating that miR-21-5p plays an inhibiting role in the progression of gliomas.

Through bioinformatics discoveries, we identified miR-21-5p binding sites in the 3'UTR of S1001A10, and the binding site between miR-21-5p and the 3'UTR of S100A10 was confirmed by double luciferase reporting experiments. Western blotting results showed that miR-21-5p overexpression significantly decreased the expression of S100A10 protein, and inhibition of miR-21-5p expression significantly increased the expression level of S100A10 protein in U87 and U251 cell lines. It was further proved that miR-21-5p targeting S100A10 can negatively regulate the expression of S100A10 in glioma.

To further confirm that miR-21-5p regulates glioma cells by targeting S100A10. siRNA of S100A10 was used to reverse the expression of S100A10 which was increased by miR-21-5p inhibitor. Transwell invasion assay and flow cytometry assay showed that decreasing the expression of S100A10 significantly reversed the promoting effect of miR-21-5p inhibitor on the invasion of U87 and U251 cells. The inhibitory effect on apoptosis was also decreased. This suggests that S100A10 is regulated by miR-21-5p in glioma progression.

## Conclusion

S100A10 is highly expressed in glioma, and down-regulation of S100A10 expression can inhibit the proliferation, invasion, and migration of glioma cells. miR-21-5p targets S100A10 and negatively regulated the proliferation, invasion, and apoptosis of glioma cells. Together, these results provide new insights into the pathogenesis of gliomas and suggest that miR-21-5p /S100A10 axis may serve as a valuable therapeutic target for glioma therapy.

## Figures and Tables

**Table 1:** Clinic parameters of enrolled patients from the TCGA database

Variables		S100A10 expression		Log-rank *P*/* P* value
		Low	High	
		180	338	0.0174^b^
Gender	Male	130	125	0.5990^ b^
	Female	128	135	
Age at initial pathologic diagnosis(year)	≥60	121	132	0.4520^ a^
	<60	118	147	
				
PTEN	Wt	31	49	0.0004^ a^
	del	113	326	0.5800^ a^
				
MYCN	Wt	123	348	0.0016^ a^
	Amp	13	21	0.1720^ a^
				
MGMT	Unmethy	55	121	0.2921^ a^
	Methy	54	115	0.0047^ a^
				
EGFR	Wt	29	37	0.0100^ a^
	Amp	111	331	0.0242^ a^
				

a: Log-rank *P*; b: *P* value

## Abbreviations

IHC: immunohistochemistry
CAL1L: Calpactin I, light chain
CLP11: Calpactin I, P11 subunit
ANX2LG: Annexin II Ligand
MMPs: matrix metalloproteinase
ECM: extracellular matrix
qPCR: quantitative polymerase chain reaction
TCGA: The Cancer Genome Atlas
EGFR: epidermal growth factor receptor
MGMT: O6-methylguanine (O6-MeG)-DNA methyltransferase
PTEN: phosphatase and tensin homolog deleted on chromosome 10
DAB: 3-diaminobenzidine
OD: optical density
ECL: enhanced chemiluminescence
